# The clinical and prognostic value of CXCL8 in cervical carcinoma patients: immunohistochemical analysis

**DOI:** 10.1042/BSR20171021

**Published:** 2017-10-06

**Authors:** Ruiling Yan, Hanlin Shuai, Xin Luo, Xueqin Wang, Baozhang Guan

**Affiliations:** 1 Fetal Medicine Department, The First Affiliated Hospital of Jinan University, No.613 Huangpu Avenue West, Tianhe District, Guangzhou 510630, Guangdong, China; 2 Department of Gynecology and Obstetrics, The First Affiliated Hospital of Jinan University, No.613 Huangpu Avenue West, Tianhe District, Guangzhou 510630, Guangdong, China; 3 Department of Nephrology, The First Affiliated Hospital of Jinan University, No.613 Huangpu Avenue West, Tianhe District, Guangzhou 510630, Guangdong, China

**Keywords:** CXCL8, cervical carcinoma, oncogene, prognosis, tumor biomarker

## Abstract

Cysteine-X-cysteine ligand 8 (CXCL8) was originally discovered as a proinflammatory chemokine. Recently, CXCL8 has been shown to act as an oncogene in several types of human cancers. However, the clinical and prognostic significance of CXCL8 in cervical cancer is poorly understood. In our study, we found that CXCL8 was highly expressed in cervical cancer tissues compared with normal cervical tissues in microarray datasets (GSE9750 and GSE7803). *CXCL8* mRNA and protein expressions were increased in cervical cancer tissues and cell lines compared with normal cervical tissues and cervical epithelial cell lines. CXCL8 protein expression was significantly correlated with clinical stage, distant metastasis, histological type, and histological grade. CXCL8 high expression was a poor independent prognostic parameter for cervical cancer patients. In conclusion, CXCL8 is highly expressed in cervical cancer tissues and cell lines, and correlated with malignant status and prognosis in cervical cancer patients.

## Introduction

Cervical cancer is the fourth most frequent malignant tumor amongst women worldwide, with an estimated 527600 new cases and 265700 deaths in 2012 [1]. Cervical cancer is more common in developing countries, which accounts for 90% cases worldwide [1]. In China, a substantially increasing incidence trend was observed, which is contrary to developed countries [2]. According to recently published data, cervical cancer is the seventh most common cancer in women with an estimated 98900 new cases and 30500 deaths in China on 2015 [2]. Based on 2017 Cancer Statistics of United States, it is estimated that approximately 12820 new cases of cervical cancer were diagnosed in 2017 in the United States, which led to 4210 deaths [3]. Nowadays, operation or radiotherapy combined with chemotherapy is a major treatment for cervical cancer patients [4,5]. Effective target therapy is destitute in cervical cancer [6,7]. Therefore, it is necessary to explore novel biomarkers for predicting clinical outcome and developing therapeutic target in cervical cancer patients.

Cysteine-X-cysteine ligand 8 (CXCL8) is a proinflammatory chemokine that was originally discovered for its role in promoting chemotaxis and degranulation of neutrophils [8]. In recent decades, CXCL8 has been suggested to serve as a multifunctional cytokine to regulate tumor cells proliferation, invasion, and migration [9]. In lung cancer and colorectal cancer, high levels of CXCL8 expression suggested an increased risk of cancer and unfavorable prognosis [10,11]. In breast cancer patients, CXCL8 overexpression was observed in tumor tissues and associated with bone metastasis [12,13]. However, the clinical and prognostic significance of CXCL8 in cervical cancer is still unknown. The aim of our study was to explore the status of CXCL8 expression in cervical cancer tissues and cell lines, and analyze the association between CXCL8 expression and clinicopathological characteristics in cervical cancer patients.

## Materials and methods

### Analysis of microarray data

Microarray dataset (GEO accession number: GSE9750) included normal cervical tissues and cervical cancer tissues, which was submitted by Murty Vundavalli on 03/12/2007 (e-mail: vvm2@columbia.edu). Microarray dataset (GEO accession number: GSE7803) included normal cervical tissues, cervical intraepithelial lesions tissues, and cervical cancer tissues, which was submitted by Rork Kuick on 15/05/2007 (e-mail: rork@umich.edu).

### Ethics statement

The present study was approved by the Research Ethics Committee of The First Affiliated Hospital of Jinan University. Informed written consents were collected from all the eligible patients and the entire study was performed based on the Declaration of Helsinki.

### Clinical samples

A total of 108 cervical cancer tissue samples and 25 normal cervical tissue samples were collected at the First Affiliated Hospital of Jinan University between January 2005 and December 2015. Clinical samples were respectively stored in liquid nitrogen for qRT-PCR and formaldehyde solution for immunohistochemistry. Clinical staging and system treatment were based on the seventh edition of AJCC Cancer Staging Manual and NCCN guideline, respectively. None of patients in the present study had received neoadjuvant antitumor treatment.

### Cell lines

Human cervical squamous cell carcinoma cell line (Caski), human cervical adenocarcinoma cell line (HeLa), human normal ectocervical cell line (Ect1/E6E7), and human normal endocervical cell line (End1/E6E7) were obtained from the Cell Bank of Type Culture Collection of the Chinese Academy of Sciences. All cells were cultured in Dulbecco’s modified Eagle’s medium (DMEM, Gibco), 100 U/ml penicillin, and 100 μg/ml streptomycin at 37°C in a humidified atmosphere with 5% CO_2_.

### Quantitative real-time PCR

Total RNA was extracted by using RNAiso Plus (Takara), according to the manufacturer’s instructions. A total of 500 ng RNA was converted into cDNA using PrimeScript™ RT reagent Kit with gDNA Eraser (Takara). Real-time PCR was performed using SYBR Green (TaKaRa) and Light Cycler Roche 480 PCR instrument. The primers are as follows: CXCL8, forward primer: 5′-AAATTTGGGGTGGAAAGGTT-3′; reverse primer: 5′-TCCTGATTTCTGCAGCTCTGT-3′. β-actin, forward primer: 5′-CCAACCGCGAGAAGATGA-3′; reverse primer: 5′-CCAGAGGCGTACAGGGATAG-3′. β-actin was used as an internal control. The relative expression was analyzed by the 2^–Δ*C*_t_^ method.

### Western blot

Total protein was extracted using cell lysis buffer (Beyotime) for Western blot. Equal amounts of protein were denatured and then separated by SDS/PAGE (12% gel). The target proteins were incubated with the following primary antibodies: CXCL8 (Abcam) or β-actin antibody (CWBio). Then the proteins were incubated with homologous secondary antibodies (CWBio). For HRP detection, an ECL chemiluminescence kit (CWBio) was used. Intensity of blots was performed by Quantity One Software (Bio–Rad).

### Immunohistochemistry

Immunohistochemical analysis was performed to measure CXCL8 protein expression in cervical cancer tissue samples. In brief, slides were baked at 60°C for 1 h, followed by deparaffinization with xylene, and rehydrated. The sections were submerged in EDTA antigenic retrieval buffer and microwaved for antigen retrieval. They were then treated with 3% hydrogen peroxide in methanol to quench endogenous peroxidase activity, followed by incubation with 5% BSA to block nonspecific binding. Sections were incubated with anti-CXCL8 (1:200 dilution, Abcam) overnight at 4°C. After washing, tissue sections were treated with secondary antibody, followed by incubation with conjugated horseradish peroxidase streptavidin. Tissue sections were then counterstained with Hematoxylin, dehydrated, and mounted. Finally, sections were viewed under a bright-field microscope.

### Evaluation of staining

The tissue sections stained immunohistochemically for CXCL8 were reviewed, and scored separately by two pathologists blinded to the clinical parameters. Any disagreements were arbitrated by a third pathologist. For CXCL8 assessment [14], this was determined by a combined score comprising the percentage of cells with staining (0, 0%; 1, 1–10%; 2, 10–50%; 3, 51–80%; 4, greater than 85% positive cells) and the intensity of the staining (0, negative; 1, weak; 2, moderate; 3, strong). The final score was calculated by multiplication of these two variables. Low expression of CXCL8 was defined as 0–4 score; high expression of CXCL8 was defined as more than 4 score.

### The Cancer Genome Atlas database analysis

The OncoLnc database (http://www.oncolnc.org/) was used to analyze the prognostic significance of CXCL8 in cervical squamous cell carcinoma and endocervical adenocarcinoma patients. The cervical squamous cell carcinoma and endocervical adenocarcinoma patient’s cohort included 264 cases. The median value of CXCL8 expression was selected as the cutoff of the high and low CXCL8 groups.

### Statistical analysis

The difference of *CXCL8* mRNA expression between cervical cancer tissues and paired adjacent normal cervical tissues was detected by the Wilcoxon signed-rank test. Student’s *t* test was used for comparisons of two independent groups. The association between CXCL8 protein expression and clinicopathologic parameter of cervical cancer patients was analyzed by chi-square test. Kaplan–Meier method was used to conduct survival analysis. Cox regression was used for univariate analysis. The significance of survival variables (*P*<0.05) in univariate analysis were included into the final multivariable Cox proportional hazards model. *P*-values in all the experiments were considered statistically significant at less than or equal to 0.05.

## Results

### CXCL8 is highly expressed in cervical cancer tissues

**Figure 1:**
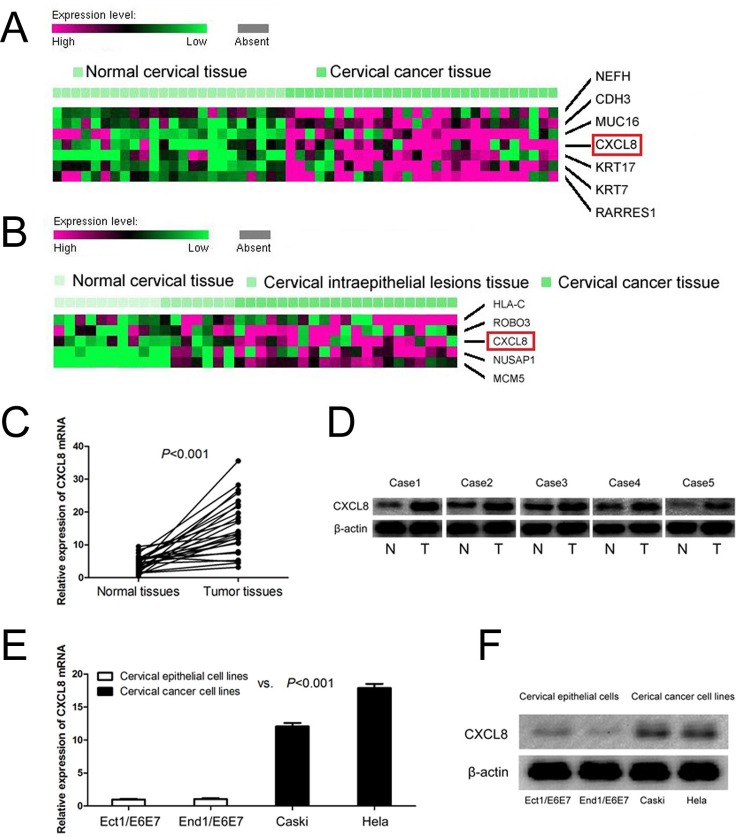
*CXCL8* mRNA and protein expressions in cervical cancer tissues and cell lines (**A**) CXCL8 high expression is observed in cervical cancer tissues compared with normal cervical tissues (GSE9750). (**B**) CXCL8 is overexpressed in cervical cancer tissues compared with normal cervical tissues and cervical intraepithelial lesions’ tissues (GSE7803). (**C**) *CXCL8* mRNA expression is higher in cervical cancer tissues than adjacent normal cervical tissues. (**D**) CXCL8 protein expression is higher in cervical cancer tissues than adjacent normal cervical tissues. (**E**) Levels of *CXCL8* mRNA are increased in cervical cancer cell lines compared with normal cervical epithelial cells lines. (**F**) CXCL8 protein expression is highly expressed in cervical cancer cell lines compared with normal cervical epithelial cell lines.

In order to explore the status of CXCL8 in cervical cancer tissues, we analyzed microarray datasets (GEO accession number: GSE9750 and GSE7803). We found that CXCL8 was highly expressed in cervical cancer tissues compared with normal cervical tissues in microarray dataset (GSE9750, Figure 1A), and also overexpressed in cervical cancer tissues compared with normal cervical tissues and cervical intraepithelial lesions’ tissues in microarray dataset (GSE7803, Figure 1B). Furthermore, we conducted qRT-PCR, Western blot, and immunohistochemistry to determine *CXCL8* mRNA and protein levels in normal cervical tissues and cervical cancer tissues. Compared with adjacent normal cervical tissues, *CXCL8* mRNA was highly expressed in cervical cancer tissues (*P*<0.001, Figure 1C). Meanwhile, Western blot suggested that CXCL8 protein expression was elevated in cervical cancer tissues compared with adjacent normal cervical tissues (Figure 1D). Immunohistochemical analysis showed that CXCL8 protein expression was increased in cervical cancer tissues (56.5%, 61/108) compared with normal cervical tissues (29.6%, 8/27) (*P*=0.013, Table 1).

**Table 1:** CXCL8 protein expression in normal cervical tissues and cervical cancer tissues

Group	*n*	CXCL8 protein expression		*P*
		High (%)	Low (%)	
Normal	27	8(29.6)	19(70.4)	0.013
Tumor	108	61(56.5)	47(43.5)	

### CXCL8 is overexpressed in cervical cancer cell lines

*CXCL8* mRNA and protein expressions were also observed in human cervical cancer cell lines (Caski and HeLa) and human normal cervical epithelial cell lines (Ect1/E6E7 and End1/E6E7). We found that levels of *CXCL8* mRNA were increased in cervical cancer cell lines compared with normal cervical epithelial cell lines (*P*<0.001, Figure 1E). Similarly, CXCL8 protein expression was shown to be overexpressed in human cervical cancer cell lines compared with normal cervical epithelial cells lines (Figure 1F).

### Correlation between CXCL8 protein expression and clinicopathological characteristics in cervical cancer patients

**Figure 2:**
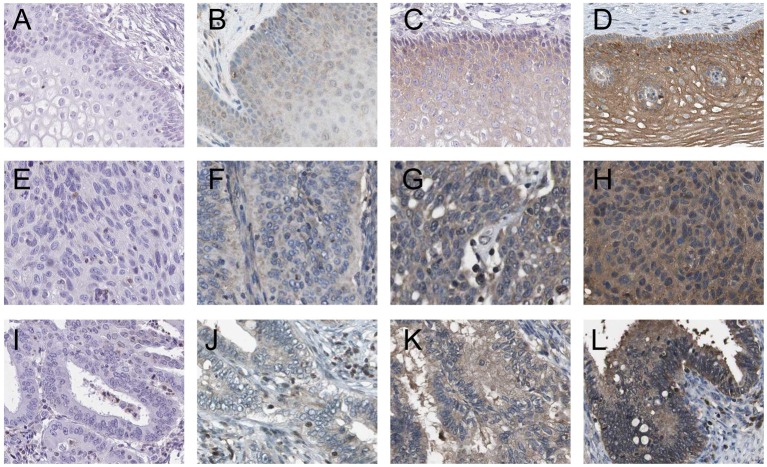
Immunohistochemical staining of CXCL8 in cervical cancer tissues (**A**) Negative expression of CXCL8 in normal cervical tissue; (**B**) weak expression of CXCL8 in normal cervical tissue; (**C**) moderate expression of CXCL8 in normal cervical tissue; (**D**) strong expression of CXCL8 in normal cervical tissue; (**E**) negative expression of CXCL8 in cervical squamous cell carcinoma tissue; (**F**) weak expression of CXCL8 in cervical squamous cell carcinoma tissue; (**G**) moderate expression of CXCL8 in cervical squamous cell carcinoma tissue; (**H**) strong expression of CXCL8 in cervical squamous cell carcinoma tissue; (**I**) negative expression of CXCL8 in cervical adenocarcinoma tissue; (**J**) weak expression of CXCL8 in cervical adenocarcinoma tissue; (**K**) moderate expression of CXCL8 in cervical adenocarcinoma tissue; (**L**) strong expression of CXCL8 in cervical adenocarcinoma tissue.

We measured the levels of CXCL8 protein expression in 108 cervical cancer samples by using immunohistochemical staining (Figure 2A–L), and analyzed the correlation between the protein expression of CXCL8 and clinicopathological characteristics of cervical cancer. As summarized in Table 2, CXCL8 protein expression was significantly correlated with clinical stage (I–IIA compared with IIB–IV; *P*=0.009), distant metastasis (absent compared with present; *P*=0.045), histological type (adenocarcinoma compared with squamous cell carcinoma; *P*<0.001), and histological grade (well compared with moderately/poorly; *P*<0.001). However, CXCL8 protein expression was not correlated with age (≤50 years compared with >50 years, *P*=0.527), tumor size (≤4 cm compared with >4 cm, *P*=0.812), and lymph node metastasis (absent compared with present; *P*=0.282).

**Table 2:** Association between CXCL8 protein expression and clinicopathological characteristics in cervical cancer patients

Characteristics	*n*	CXCL8 protein expression		*P*
		High (%)	Low (%)	
Age (years)				
≤50	52	31 (59.6)	21 (40.4)	0.527
>50	56	30 (53.6)	26 (46.4)	
Clinical stage				
I–IIA	49	21 (42.9)	28 (57.1)	0.009
IIB–IV	59	40 (67.8)	19 (32.2)	
Tumor size (cm)				
≤4	68	39 (57.4)	29 (42.6)	0.812
>4	40	22 (55.0)	18 (45.0)	
Lymph node metastasis				
Absent	65	34 (52.3)	31 (47.7)	0.282
Present	43	27 (62.8)	16 (37.2)	
Distant metastasis				
Absent	101	54 (53.5)	47 (46.5)	0.045
Present	7	7 (100)	0 (0)	
Histological type				
Adenocarcinoma	18	18 (100)	0 (0)	<0.001
Squamous cell carcinoma	90	43 (47.8)	47 (52.2)	
Histological grade				
Well	49	18 (36.7)	31 (63.3)	<0.001
Moderately/poorly	59	43 (72.9)	16 (27.1)	

### CXCL8 high expression is a poor independent prognostic parameter for cervical cancer patients

**Figure 3:**
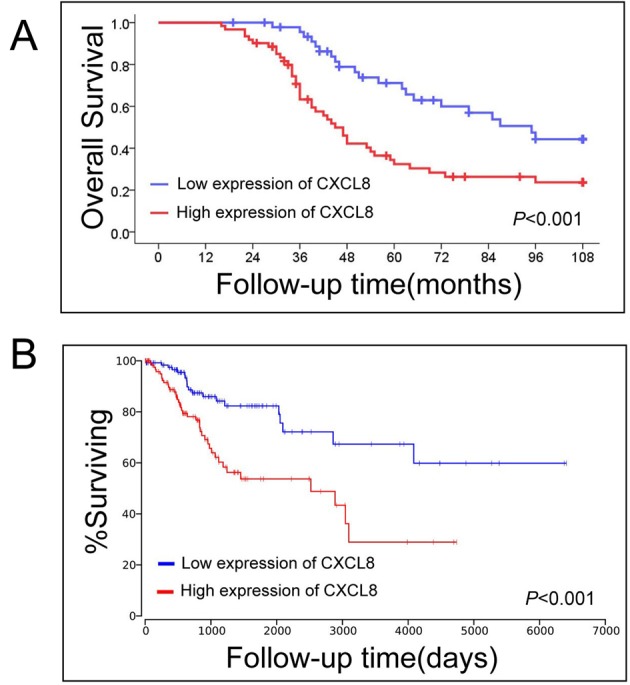
The prognostic significance of CXCL8 in cervical cancer (**A**) CXCL8 high expression is negatively associated with overall survival in 108 cervical cancer patients from our study. (**B**) The Cancer Genome Atlas database including 246 cervical cancer patients shows that CXCL8 protein’s high expression had shorter overall survival compared in patients with CXCL8 protein’s low expression.

The prognostic value of CXCL8 protein was further identified in cervical cancer patients. Kaplan–Meier survival analysis indicated that cervical cancer patients with CXCL8 protein’s high expression had shorter overall survival compared in patients with CXCL8 protein low expression (*P*<0.001, Figure 3A). Regardless of clinical stage, lymph node metastasis, distant metastasis, histological type and histological grade, we found that CXCL8 protein high expression also served as a poor prognostic parameter in cervical cancer patients through univariate Cox regression analyses. Meanwhile, multivariate Cox regression analyses suggested that CXCL8 protein’s high expression was an unfavorable independent prognostic factor for cervical cancer patients (*P*=0.030, Table 3).

**Table 3:** Summary of univariate and multivariate Cox regression analysis of overall survival duration in cervical cancer patients

Parameter	Univariate analysis			Multivariate analysis		
	*P*	HR	95% CI	*P*	HR	95% CI
Age (years)	0.490	0.838	0.507–1.385	0.153	0.663	0.377–1.165
(≤50 compared with >50)						
Clinical stage	<0.001	2.645	1.546–4.526	0.289	1.557	0.687–3.528
(I-IIA vs. IIB-IV)						
Tumor size (cm)	0.237	1.356	0.819–2.247	0.303	1.342	0.767–2.349
(≤4 compared with >4)						
Lymph node metastasis	<0.001	3.221	1.839–5.639	0.031	2.452	1.084–5.546
(Absent compared withpresent)						
Distant metastasis	<0.001	5.217	2.108–12.910	0.153	2.365	0.726–7.700
(Absent compared with present)						
Histological type	<0.001	0.331	0.179-0.611	0.681	1.198	0.507-2.832
(Squamous cell carcinoma compared with adenocarcinoma)						
Histological grade	0.003	2.209	1.313–3.717	0.166	1.499	0.846–2.659
(Well compared with moderately/poorly)						
CXCL8 protein expression	0.002	2.338	1.378–3.967	0.030	1.917	1.067–3.445
(Low compared with high)						

Abbreviations: HR, hazard ratio; 95% CI, 95% confidence interval.

In order to confirm the prognostic significance of CXCL8 in cervical cancer patients, we analyzed a cohort that included 246 cervical squamous cell carcinoma and endocervical adenocarcinoma patients from The Cancer Genome Atlas database. Similar to our result, we also found cervical cancer patients with CXCL8 high expression had a shorter survival time that those with CXCL8 low expression (*P*<0.001, Figure 3B).

## Discussion

CXCL8 is a prototypical chemokine which belongs to the CXC family [15]. The gene encoding CXCL8 is located on chromosome 4q13-q21 [16]. The function of CXCL8 is mainly based on its interaction with G-protein-coupled receptors cysteine-X-cysteine chemokine receptors (CXCR1 and CXCR2) [17]. In recent decades, the mechanism of CXCL8-CXCR1/2 signaling in regulating regulate tumor cells proliferation, invasion, and migration has been explored extensively [18,19].

High expression of CXCL8 was observed in several types of human cancer, such as lung cancer [20,21], breast cancer [12,13,22], colorectal cancer [14,23–25], prostate cancer [26], pancreatic cancer [27], hepatocellular carcinoma [28], head and neck squamous cell carcinoma [29], gastric cancer [30], leukemia [31], and melanoma [32–34]. The status of CXCL8 expression in cervical cancer was still unknown. In our study, we observed that CXCL8 was highly expressed in cervical cancer tissues compared with normal cervical tissues in microarray datasets. Furthermore, we confirmed that *CXCL8* mRNA was highly expressed in cervical cancer tissues and cell lines compared with adjacent normal cervical tissues and cervical cancer cell lines. Meanwhile, we measured the levels of CXCL8 protein expression in 108 cervical cancer samples by using immunohistochemical staining, and analyzed the correlation between the protein expression of CXCL8 and clinicopathological characteristics of cervical cancer. We found that CXCL8 protein expression was significantly correlated with clinical stage, distant metastasis, histological type and histological grade. In colorectal cancer, Cheng et al. found that CXCL8 expression was obviously higher in patients with stage T3 or T4, lymph node metastasis, and liver metastasis [23,24]. Similarly, Li et al. [28] reported that CXCL8 expression was higher in hepatocellular carcinoma samples with vascular invasion, intrahepatic, distant metastasis, and higher TNM stage. In prostate cancer patients, Uehara et al. [35] suggested that CXCL8 high expression was positively correlated with Gleason score and pathologic stage of tumors. Scheibenbogen et al. [32] showed CXCL8 overexpression was positively associated with tumor load in melanoma patients. The above-mentioned studies consistently showed CXCL8 overexpression was correlated with the malignant status in human cancer, suggesting that CXCL8 overexpression may serve as unfavorable prognostic biomarker for human cancer patients.

Recent studies showed that CXCL8 high expression serves as an unfavorable prognostic factor in most human cancers, such as breast cancer [22], colorectal cancer [14,23,24], melanoma [32], and lung cancer [36,37]. In breast cancer patients, Bieche et al. [22] found that high expression level of CXCL8 correlated with significantly shorter relapse-free survival. Several studies showed CXCL8 overexpression obviously associated with poor overall and disease-free survival in colorectal cancer patients [14,23,24]. In non-small-cell lung cancer patients, Yuan et al. [36] suggested that survival and post-operative relapse time were evidently shorter in patients with CXCL8 high expression than in those with CXCL8 low expression. Furthermore, Sunaga et al. [37] indicated that lung adenocarcinoma patients with CXCL8 high expression showed markedly shorter disease-free survival and overall survival than those with CXCL8 low expression. However, Li et al. [28] reported that there was no obvious difference between hepatocellular carcinoma patients with high and low expression of CXCL8 group regarding disease-free survival or overall survival. The discrepancy in Li et al. [28] data would most likely to be due to the heterogenicity in human cancers. The prognostic significance of CXCL8 was unclear in cervical cancer patients. In our study, we observed cervical cancer patients with CXCL8 protein high expression had shorter overall survival compared with patients with CXCL8 protein low expression. Furthermore, we analyzed a cohort that included 246 cervical cancer patients from The Cancer Genome Atlas database, and found that patients with high level of CXCL8 had shorter overall survival compared with patients with low level of CXCL8, which was consistent with our result. Overall, our study demonstrated that CXCL8 expression was significantly increased in cervical cancer and associated with the malignant status and prognosis in cervical cancer patients. However, due to the limited sample size of patients in our study, further studies would be needed to verify these findings and establish the role of CXCL8 as a reliable clinical predictor for the outcome of cervical cancer patients.

In recent decades, CXCL8 signaling pathway inhibitors have been considered to be anticancer drug candidates. Reparixin (also known as repertaxin), a small molecule inhibitor, prevents CXCL8 from binding and interacting with its receptors by keeping both CXCR1 and CXCR2 in an inactive conformation [38]. An open label Phase I clinical trial including 33 metastatic breast cancer patients was conducted to detect the pharmacokinetic profile and evaluate safety and tolerability of orally administered reparixin in combination with a fixed dose of weekly paclitaxel (NCT02001974). Subsequently, reparixin has been utilized in a double-blind Phase II study with 190 estimated enrolments is in progress to compare the progression-free survival of metastatic triple negative breast cancer patients receiving paclitaxel alone or with reparixin (NCT02370238). Moreover, HuMab10F8 (also known as HuMax-CXCL8), a specific antibody, blocks CXCL8 signaling by neutralizing CXCL8 [39]. A Phase I clinical trial to perform gradient trial with HuMax-CXCL8 is recruiting patients with metastatic or unresectable, locally advanced malignant solid tumors (NCT02536469). Due to cervical cancer patients with CXCL8 overexpression, CXCL8 signaling pathway inhibitors may have favorable effectiveness for cervical cancer patients.

In conclusion, CXCL8 is overexpressed in cervical cancer tissues and cell lines, and is associated with malignant status and prognosis in cervical cancer patients.

## Abbreviation

CXCL8: cysteine-X-cysteine ligand 8
GEO: gene expression omnibus
